# Lentivirus-mediated downregulation of α-synuclein reduces neuroinflammation and promotes functional recovery in rats with spinal cord injury

**DOI:** 10.1186/s12974-019-1658-2

**Published:** 2019-12-30

**Authors:** Hong Zeng, Nan Liu, Yan-yan Yang, Hua-yi Xing, Xiao-xie Liu, Fang Li, Gao-yan La, Meng-jie Huang, Mou-wang Zhou

**Affiliations:** Department of Rehabilitation Medicine, Peking University Third Hospital, 49 North Garden Road, Beijing, 100191 China

**Keywords:** Spinal cord injury, α-Synuclein, Microglia, Astrocyte, Blood-cerebrospinal barrier, Matrix metalloproteinase

## Abstract

**Background:**

The prognosis of spinal cord injury (SCI) is closely related to secondary injury, which is dominated by neuroinflammation. There is evidence that α-synuclein aggregates after SCI and that inhibition of α-synuclein aggregation can improve the survival of neurons after SCI, but the mechanism is still unclear. This study was designed to investigate the effects of α-synuclein on neuroinflammation after SCI and to determine the underlying mechanisms.

**Method:**

A T3 spinal cord contusion model was established in adult male Sprague-Dawley rats. An SNCA-shRNA-carrying lentivirus (LV-SNCA-shRNA) was injected into the injury site to block the expression of α-synuclein (forming the SCI+KD group), and the SCI and sham groups were injected with an empty vector. Basso-Beattie-Bresnahan (BBB) behavioural scores and footprint analysis were used to detect motor function. Inflammatory infiltration and myelin loss were measured in the spinal cord tissues of each group by haematoxylin-eosin (HE) and Luxol Fast Blue (LFB) staining, respectively. Immunohistochemistry, Western blot analysis, and RT-qPCR were used to analyse protein expression and transcription levels in the tissues. Immunofluorescence was used to determine the morphology and function of glial cells and the expression of matrix metalloproteinase-9 in the central canal of the spinal cord. Finally, peripheral serum cytokine levels were determined by enzyme-linked immunosorbent assay.

**Results:**

Compared with the SCI group, the SCI+KD group exhibited reduced inflammatory infiltration, preserved myelin, and functional recovery. Specifically, the early arrest of α-synuclein inhibited the pro-inflammatory factors IL-1β, TNF-α, and IL-2 and increased the expression of the anti-inflammatory factors IL-10, TGF-β, and IL-4. The neuroinflammatory response was regulated by reduced proliferation of Iba1+ microglia/macrophages and promotion of the shift of M1-polarized Iba1+/iNOS+ microglia/macrophages to M2-polarized Iba1+/Arg1+ microglia/macrophages after injury. In addition, compared with the SCI group, the SCI+KD group also exhibited a smaller microglia/astrocyte (Iba1/GFAP) immunostaining area in the central canal, lower MMP-9 expression, and improved cerebrospinal barrier function.

**Conclusion:**

Lentivirus-mediated downregulation of α-synuclein reduces neuroinflammation, improves blood-cerebrospinal barrier function, promotes functional recovery, reduces microglial activation, and promotes the polarization of M1 microglia/macrophages to an M2 phenotype to confer a neuroprotective immune microenvironment in rats with SCI.

## Introduction

Traumatic spinal cord injury (SCI) is caused by an external force applied directly or indirectly to the spinal cord. Neuronal loss, axonal degeneration, and demyelination at the injury site cause functional or organic changes in the spinal cord. Although modern medical rehabilitation has improved patient outcomes and prognosis, drug therapies to prevent neuronal death or promote regeneration remain limited [1, 2]. Studies have shown that in the context of SCI, mechanical damage causes neurons and glia at the injury site to die within minutes to hours. This death is followed by a delayed secondary injury phase in which the characteristic neuroinflammatory response persists. SCI-induced glial activation and the subsequent release of inflammatory factors such as interleukin (IL), tumour necrosis factor (TNF), and interferon (IFN) accelerate neuronal death while inducing vascular endothelial cells to express multiple cell adhesion and chemotaxis molecules to attract more inflammatory factors. These factors also typically stimulate nitric oxide (NO) release, increase capillary permeability, and cause blood-cerebrospinal barrier (BCSB) dysfunction [3, 4]. SCI triggers a range of cellular and molecular events, including microglial/astrocyte activation, peripheral blood-derived macrophage infiltration, pro-inflammatory/anti-inflammatory response imbalance, abnormal mitochondrial activity, oxidative stress, abnormal protein aggregation, and free radical toxicity. These processes induce neuronal death and lead to permanent neurological deficits. The amplification of the inflammatory response is the main cause of secondary injury, and the secondary injury process worsens outcomes. Therefore, improving the immune environment of the spinal cord during secondary injury is the most urgent and important goal of treatment [5].

Neuroinflammation originates from innate immunity of the central nervous system (CNS). Microglia are the most common immune cells in the CNS. Under normal conditions, microglia are in a quiescent state; however, after injury or infection, microglia are activated into effector cells with immune functions. At present, two forms of microglial/macrophage polarization are recognized: (1) “classic” M1 polarization, which occurs through the pro-inflammatory pathway and produces inflammatory cytokines, reactive oxygen species, and free radicals, and (2) “alternative” M2 polarization, which occurs through the anti-inflammatory pathway and is often associated with the anti-inflammatory response, tissue repair, and metabolic homeostasis [6]. An increasing number of animal experiments have found that cytokines can be modulated to alter the expression of different microglial subtypes, thereby activating different polarization pathways after nerve injury. In addition, many studies have found that reactive astrocytes in SCI models participate in the development of neuroinflammation in the acute and subacute phases; the chronic phase is closely related to scar formation after injury and hinders nerve regeneration and repair [7]. Furthermore, secondary damage involves not only upregulation of pro-inflammatory factors but also cell excitotoxicity, metabolic failure, and impairment of the clearance of protein deposits after cell death, all of which cause acute and chronic neuroinflammation [7].

α-Synuclein (α-Syn) is a soluble protein expressed in the presynaptic and perinuclear phases of nerves. Its accumulation has been associated with many degenerative neuropathies. The structure of α-Syn depends largely on the intracellular environment in which it is located, and the protein exhibits different structural forms, such as monomers, oligomers, fibrils, and fibres. Synaptic nucleoproteins aggregate easily under pathological conditions. The formation of an insoluble fibrin precipitate eventually leads to the death of nerve cells, and the aggregation of α-Syn increases mitochondrial dysfunction, oxidative stress, apoptotic pathway activation, and glial activation in the CNS [8]. SNCA, a key gene that encodes the α-Syn protein, can be inhibited by lentivirus-mediated SNCA short hairpin RNA (LV-SNCA-shRNA) [9]. Within α-Syn, the Ser-129 residue can be phosphorylated by G-coupled receptor protein kinases (GCRPs), and this phosphorylation promotes the oligomerization of α-Syn fibre filaments by altering the C-terminal potential distribution and hydrophobicity of α-Syn [10]. α-Syn that is phosphorylated at the Ser-129 site is widely and selectively distributed in many synaptic pathological lesions, such as Lewy bodies [10]. Studies have shown that after SCI, α-Syn, knockdown of which inhibits neuronal apoptosis and promotes spontaneous repair, forms punctate aggregates in spinal cord neurons [11]. Another report has shown that α-Syn triggers the migration of microglia after SCI [12]. However, the effect of α-Syn on neuroinflammation after SCI and its mechanism remains unclear.

Therefore, the goal of this study was to observe α-Syn expression and Ser-129 phosphorylation after SCI in vivo. The effects of α-Syn on neuroinflammation-related factors, including microglial/astrocyte activation, phenotype polarization, and MMP-9 expression, were detected following SCI. Furthermore, we explored the effects of α-Syn on the BCSB after injury. This study clarifies whether neuroimmunity can be improved by knocking down α-Syn.

## Materials and methods

### Animals

A total of 120 specific pathogen-free (SPF) adult male Sprague-Dawley rats (Department of Animal Science, Peking University School of Medicine, Beijing, China) were used in this study. All rats were housed in separate cages under a 12-h light-dark cycle at 23 ± 1 °C and 50% relative humidity, and food and water were available ad libitum. All rats were acclimatized to the environment for at least 1 week prior to the experiment and were maintained as directed by the experimental animal care and use guidelines. The study was approved by the Animal Welfare Ethics Branch of the Peking University Bioethics Committee.

**Fig. 1:**
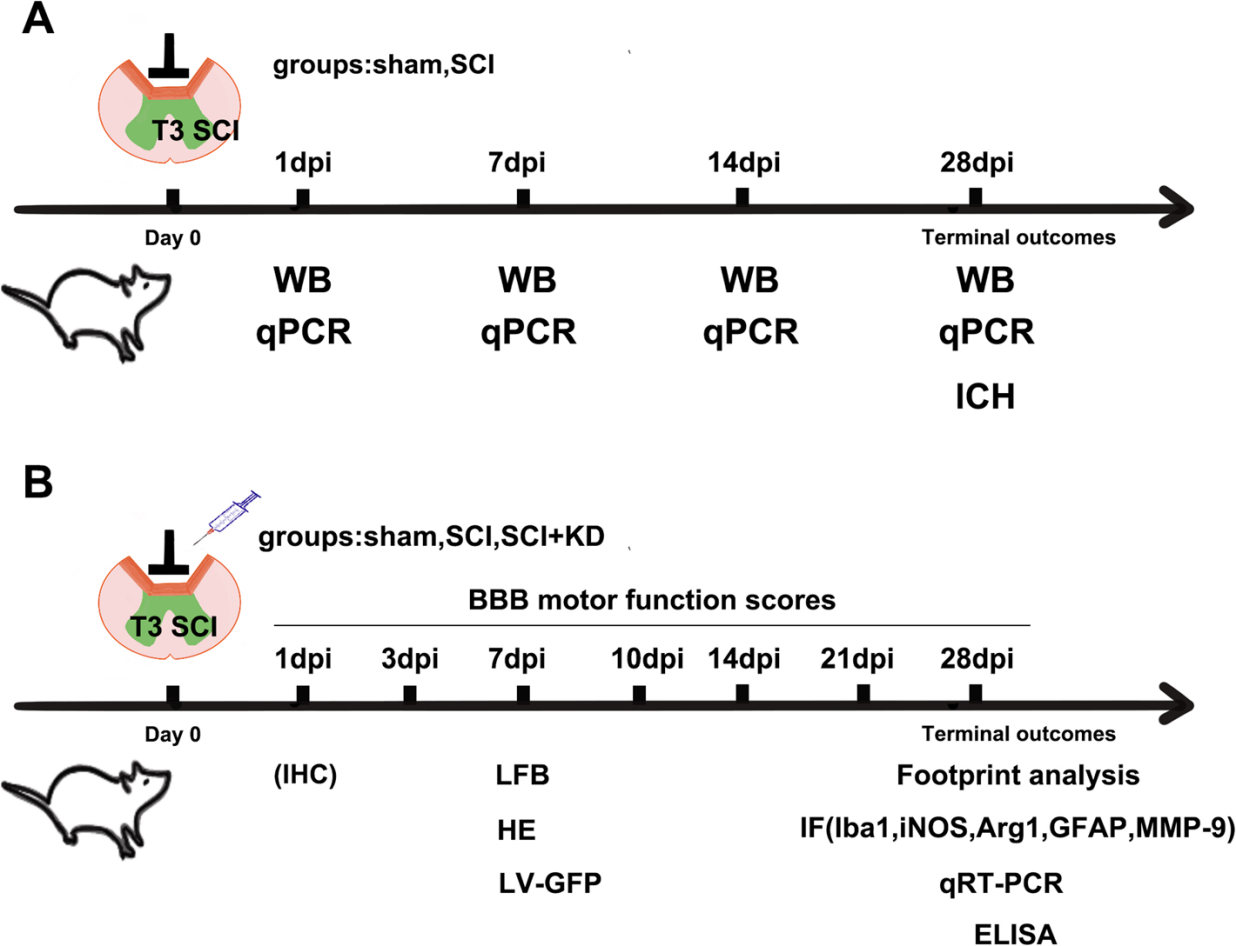
**a**, **b** Timeline of the experimental protocol

The animals were randomized into the following three groups (*n* = 40 in each group): (1) the sham+LV-pLent-U6-Puro group (the sham group); (2) the SCI+LV-pLent-U6-Puro group (the SCI group); and (3) the SCI+LV-SNCA-shRNA group (the SCI+knockdown [KD] group) (Fig. 1).

### Construction of the lentiviral LV-SNCA-shRNA vector

Lentiviruses containing SNCA-shRNA (NM_019169.2) were constructed and synthesized by ShanDong ViGene Co., Ltd. (Shandong, China). The primers for SNCA were as follows: forward, 5′-GTGGCTGCTGCTGAGAAAAC-3′ and reverse, 5′-TCCATGAACGACTCCCTCCT-3′. The virus titre of LV-SNCA-shRNA was 1.0 × 10E9 TU/ml. In addition, an LV-GFP-SNCA-shRNA green fluorescent protein (GFP)-tagged lentivirus was constructed to verify the efficiency of transfection and knockdown.

### Surgery and transfection

All rats received prophylactic antibiotic treatment with ampicillin sodium (80 mg/kg; Harbin Pharmaceutical Group Co., Ltd., Harbin, China) for 3 days before SCI surgery. The rats were intraperitoneally injected with 2% sodium pentobarbital (0.1 ml/kg) and placed in a prone position on the operating table. The limbs were fixed, and the upper chest was raised with a cotton pad. Along the T2 spine of each rat, the C8-T4 dorsal skin was dissected, the back muscle was peeled off layer by layer, and the T3 segment of the thoracic vertebra was dissected. The spinal cord was removed by performing a laminectomy of the T3 segment under a surgical microscope. In the sham group, the incision was closed layer by layer after the spinal cord was exposed, but no injury was induced. The SCI group was injured with a PSI-IH precision striking device (IH impactor; Precision Systems and Instrumentation, Lexington, KY, USA) after the spinal cord was exposed. The striking head was adjusted over the exposed T3 spinal cord segment, dropped so that it just touched the dural sac, and then raised by 2 cm. The force was set to 400 kilodynes, the compression time was set to 5 s, and the number of hits was set to one. The standards for successful generation of the rat SCI model were the presence of a contusion at the injury site, convulsions in both lower extremities, and spastic swaying of the tail. The force with which the spinal cords of the rats were impacted was monitored by a computer. Following SCI, 10 μl of lentivirus containing the target gene shRNA was injected in situ using a microsyringe. The sham group and the SCI group were given the same dose of an empty lentiviral vector. After injection, the dorsal tissue of each rat was sutured layer by layer. After the procedure, the rats were allowed to recover on a heated blanket and were placed in a clean cage for observation. Beginning immediately after surgery, the rats were injected subcutaneously with Ringer’s sodium lactate solution (5 ml) and ampicillin sodium twice daily (morning and afternoon) until the third day after injury. The bladders of the rats were squeezed 3 times daily after surgery until spontaneous urination was restored. All assessments and analyses were performed by experienced researchers who were unaware of the experimental design.

### Behavioural tests

#### Hindlimb exercise score

Basso-Beattie-Bresnahan (BBB) motor function scores were used for evaluation of hindlimb motor function [13]. The rats were placed on a circular platform with a diameter of 2 m. The walking and limb activity scores of the hindlimbs were observed and recorded. In the first stage (0–7 points), the joint activity of the hindlimbs of the animals was scored. In the second stage (8–13 points), the gait and the coordination of the hindlimbs were scored. In the third stage (14–21 points), the fine movements of the claws were judged. The scores for the three stages together totalled 21 points. Each group was scored 1 day before surgery and 1, 3, 7, 10, 14, 21, and 28 days post-injury (dpi).

#### Footprint analysis

Gait behaviour and motor coordination were assessed on the 28th day after injury [14]. The forelimbs and hind paws were coated with dyes of different colours, and the animals were placed on a 7.5 cm × 100 cm runway covered with white paper. The animals were encouraged to walk straight to the finish line so that representative images of their gaits could be obtained and coordination could be assessed.

### Tissue preparation

At the predetermined time points, each rat was anaesthetized, the diaphragm was cut, and the pericardium was opened. After blood was collected from the left apex, 5 mm of the spinal cord above and below the T3 injury site was quickly placed in a cryotube, frozen in liquid nitrogen, and stored in a − 80 °C low-temperature freezer. In addition, some rats were perfused with 150 ml of sterile 0.9% normal saline, and the right atrial appendage was cut at the same time. After the effluent liquid became clear, 300 ml of 4% paraformaldehyde (Biosharp, Beijing, China) was used for perfusion until the tissues of the rats became hard. The tissue near the injury site at the T3 level (5 mm above and below) was then removed and placed in paraformaldehyde overnight. The tissue was either dehydrated in xylene and gradient alcohol solutions, embedded in paraffin, and cut into 5-μm serial sections with a slicer or dehydrated in sucrose solutions (10%, 20%, and 30%), embedded in optimum cutting temperature (OCT) compound, cut into continuous 20-μm frozen sections with a microtome, and processed for immunofluorescence (IF).

### Paraffin section histopathological staining

At 28 dpi, paraffin sections from each group were heated at 60 °C, placed in xylene I and II for 30 min, and placed in gradient alcohol solutions of 100%, 100%, 95%, 95%, and 80% for 5 min each. The sections were rinsed twice with steamed water.

1. **HE staining**

A series of sections were stained with haematoxylin for 1 min, double-rinsed in distilled water, differentiated in 1% hydrochloric acid, double-rinsed in distilled water, and stained with eosin (Sigma-Aldrich) for 2 min.

2. **LFB staining**

A series of sections were stained with a 0.1% Luxol Fast Blue (LFB; Sigma-Aldrich) solution, sealed for 8 h at 60 °C, washed with distilled water, and placed in 95% alcohol for 10 min. Each mixture was separated in a 0.05% lithium carbonate aqueous solution (Leagene, Beijing, China) for 10 s and in a 70% alcohol solution for 20 s. The above two steps were repeated until the grey and white matter (GM and WM, respectively) were clearly observable under the microscope.

The above sections were dehydrated by a conventional alcohol gradient (80%, 95%, 95%, 100%, 100% alcohol for 2 min), placed in xylene I and II for 10 min, and then sealed with neutral gum. Images of both haematoxylin-eosin (HE) staining and LFB staining were captured with a Nano Zoomer Digital Pathology system (Hamamatsu, Japan). Three blinded experimenters used Image-Pro Plus 6.0 (Media Cybernetics, Rockville, MD, USA) to calculate the staining density to quantify the myelin and lesion area. There were five animals per group. For each animal, five spinal cross-sections in the rostral-caudal plane taken from the level of the injury were analysed.

### IHC

For immunohistochemistry (IHC), the 5-μm paraffin sections were dewaxed in water, and antigen retrieval was carried out in a pressure cooker with sodium citrate buffer (10 mM, pH 6.0; Boster Biological Technology, Ltd., Wuhan, China). The tissues were then blocked with 3% H_2_O_2_ to quench endogenous peroxidase activity. The tissues were blocked in 10% goat serum (Boster Biological Technology, Ltd) for 30 min, incubated with a rabbit anti-α-Syn antibody (1:2000; Abcam, Cambridge, MA, USA), rabbit anti-Iba1 (1:200; GeneTex, Inc., USA), and mouse anti-glial fibrillary acid protein (GFAP; 1:500; Santa Cruz, Dallas, TX) at 37 °C for 2 h, rinsed with PBS, and incubated with a goat anti-rabbit IgG secondary antibody (1:100; Zhongshan Jinqiao, Beijing, China) for 30 min. After washing, DAB (Zhongshan Jinqiao) was used for detection. The sections were then rinsed with water, the nuclei were counterstained with haematoxylin, the sections were placed in gradient alcohol solutions for 2 min each and in xylene I and II for 5 min each, and the slides were sealed with neutral resin. The sections were examined at × 20 magnification using an electron microscope (ECLIPSE 90i, Nikon, Japan) and Image-Pro Plus 6.0 to detect α-Syn staining in the GM and WM (through quantitative analysis of the mean optical density of the α-Syn+ cells). There were five animals per group. The average area of 4 different sections from each rat was calculated.

### IF

The frozen sections were thawed at room temperature for 30 min. The sections were washed three times (for 10 min each) with 0.1 mmol/l PBS containing 0.1% Triton X-100 (Sigma-Aldrich) (PBS-TX). The sections were pre-incubated in permeabilization blocking buffer (0.1 mmol/l PBS, pH 7.3, containing 0.5% Triton) for 15 min at 37 °C. The sections were then blocked with 10% (v/v) goat serum (Boster Biological Technology, Ltd) for another 30 min and incubated overnight with a primary antibody at 4 °C. After washing with PBS-TX the next day (3 times for 10 min each), the sections were incubated with a secondary antibody for 1 h at room temperature and then washed with PBS-TX. The nuclei were stained with 4,6-diamidino-2-phenylindole (DAPI, 1 μg/ml; Sigma-Aldrich) for 5 min, and the sections were washed with PBS-TX and sealed with an anti-fluorescence quencher. Images were captured under a Leica DM4 B confocal fluorescence microscope (Leica Microsystems Inc., Wetzlar, Germany) with a Leica TCS SP8 system (Leica Microsystems Inc). Negative controls were incubated with the corresponding isotype serum instead of a primary antibody.

The following primary antibodies were used at the indicated dilutions: mouse anti-ionized calcium-binding adaptor molecule (Iba1; 1:100; Abcam, Cambridge, MA), rabbit anti-Iba1 (1:200; GeneTex, Inc., USA), mouse anti- glial fibrillary acid protein (GFAP; 1:500; Santa Cruz, Dallas, TX), rabbit anti-iNOS (inducible nitric oxide synthase, 1:400; Abcam, Cambridge, MA), rabbit anti-arginase-1 (Arg1; 1:400; GeneTex, Inc., USA), and rabbit anti-matrix metalloproteinase-9 (MMP-9; 1:100; Abcam, Cambridge, MA). The following fluorescent secondary antibodies were used at the indicated dilutions: Alexa Fluor 594-conjugated AffiniPure goat anti-mouse IgG (H + L) (1:800; Jackson ImmunoResearch Laboratories, West Grove, PA), Alexa Fluor 488-conjugated AffiniPure goat anti-rabbit IgG (H + L) (1:400; Jackson ImmunoResearch Laboratories), Cy3-conjugated goat anti-rabbit IgG (H + L) (1:200; Boster Biological Technology, Ltd), and Alexa Fluor 488-conjugated AffiniPure goat anti-mouse IgG (H + L) (1:400; Jackson ImmunoResearch Laboratories).

There were 5 animals per group, and three sections from each animal at the same level (the injury level) were used to determine the number of positive cells or the mean optical density (mean optical density = integrated optical density (IOD)/area) for fluorescence quantification. The number of Iba1+ cells in each transverse section of the spinal cord was calculated by tissue fluorescence panoramic scanning at × 5 magnification. The remaining 4 random fields were observed at ×40 magnification to calculate the number of Iba1+/iNOS+ cells and the number of Iba1+/Arg1+ cells. The normalized mean fluorescence intensity of GFAP, Iba1, and MMP-9 in the central canal of the spinal cord was calculated. The experimenter who performed the analysis was unaware of the experimental groups of the rats.

### RT-qPCR

For quantitative real-time PCR (RT-qPCR), total RNA was first extracted from spinal cord tissues with TRIzol (Invitrogen, Thermo Fisher Scientific, Inc., USA) according to the manufacturer’s protocol. The ratios of absorbance at 260/280 nm and 260/230 nm were then determined using an ultraviolet-visible light spectrophotometer (NanoDrop 2000, Thermo, Waltham, MA, USA) to assess the purity and concentration of total RNA in each sample. A FastKing cDNA First Strand Synthesis Kit (Tiangen, Beijing, China) was used to synthesize cDNA from total RNA (2 μg/sample), and then RT-qPCR was carried out using SYBR Green PCR Master Mix (Tiangen, Beijing, China). The expression level of glyceraldehyde 3-phosphate dehydrogenase (GAPDH) was used as an internal control. Three replicate wells were set up for each reaction. All primers used in this experiment were supplied by Sangon Biotech Co., Ltd. (Shanghai) (Table 1 lists the primer sequences). QuantStudio Design and Analysis Software (Applied Biosystems) was used to perform the following reaction: initial denaturation at 95 °C for 15 min and 40 cycles of 95 °C for 10 s, annealing at 55 °C for 30 s and 72 °C for 32 s (for SNCA, GAPDH, IL-1β, and CD86) or 40 cycles of 95 °C for 15 s, annealing at 60 °C for 35 s, and 72 °C for 32 s (for CD206 and IL-10). Melting curve analysis confirmed the primer specificity and determined the cycle threshold (CT) fluorescence values. The data were analysed by the 2−ΔΔCT method by researchers blinded to the experimental groups of the animals.

**Table 1:** Forward and reverse primer sequences for qRT-PCR

Rat gene	Forward primer (5′-3′)	Reverse primer (5′-3′)
SNCA	FP: 5′-GTGGCTGCTGCTGAGAAAAC-3′	RP: 5′-TCCATGAACGACTCCCTCCT-3′
GAPDH	FP: 5′-GGCACAGTCAAGGCTGAGAATG-3′	RP: 5′-ATGGTGGTGAAGACGCCAGTA-3′
IL-1β	FP: 5′-AATGCCTCGTGCTGTCTGA-3′	RP: 5′-GGATTTTGTCGTTGCTTGTCTC-3′
CD86	FP 5′-GATTGCAGGTCCCAGTTCACTTC-3′	RP 5′-CCACTGTCCTGCTTGGACTCAC-3′
CD206	FP: 5′-TGGAGTGGCAGGTGGTTTATG-3′	RP: 5′-GGTTCAGGAGTTGTTGTGGGC-3′
IL-10	FP: 5′-CAGACCCACATGCTCCGAGA-3′	RP: 5′-CAAGGCTTGGCAACCCAAGTA-3′

### Western blotting

Total protein was extracted using radioimmunoprecipitation assay (RIPA) lysis buffer (including protease inhibitor cocktail) [15]. After measuring the protein concentration using a BCA assay kit (Thermo Scientific, MA, USA), the protein was subjected to Western blotting. Equal amounts of protein from each sample were separated using 8%, 15% SDS-PAGE and transferred to polyvinylidene fluoride membranes (Merck Millipore, Billerica, MA, USA). The membranes were blocked with 5% skim milk at room temperature (22–25 °C) for 1 h and then incubated with a monoclonal rabbit anti-α-Syn antibody (1:10000, Abcam), a polyclonal mouse anti-α-Syn antibody (1:500, BD Biosciences), and a rabbit anti-α-Syn primary antibody (phospho-S129, 1:1000, Abcam) or a rabbit anti-β-actin antibody (1:10000; Abcam, Cambridge, UK) overnight at 4 °C. After incubation with the corresponding secondary antibodies for 1 h, the membranes were scanned using an Odyssey Sa imaging system (LI-COR Biosciences, Lincoln, NE, USA). The density of the results was quantified by two experimenters using ImageJ software (NIH, Bethesda, MD, USA), and the two experimenters were blinded to the characteristics of the samples studied.

### ELISA

Serum samples were prepared by centrifugation using coagulant blood collection tubes. Enzyme-linked immunosorbent assay (ELISA) kits were used to measure the serum levels of TNF-α, IL-1β, IL-2, IL-10, IL-4, transforming growth factor-β (TGF-β) (Boster Biological Technology, Ltd., Wuhan, China), and IFN gamma (IFN-γ, R&D Systems, Minneapolis, MN) according to the manufacturer’s instructions on the 28th day after surgery. The absorbance was measured at 450 nm with a Multi-Mode Microplate Reader (Varioskan Flash, Thermo Scientific Inc., USA). The concentrations of TNF-α, IL-1β, IL-2, IL-10, IL-4, TGF-β, and IFN-γ were calculated from standard curves and are expressed in pg/ml.

### Statistical analysis

The data are expressed as the mean ± standard deviation (SD). Statistical analysis was performed in GraphPad Prism 7.0 (GraphPad Software Inc., San Diego, CA). Comparisons between the two groups were performed using Student’s *t* test or the Mann-Whitney test, as appropriate. One-way and two-way analysis of variance (ANOVA) and Tukey’s test for multiple comparisons were used to analyse differences between groups. BBB motor function scores were analysed by two-way repeated-measures ANOVA followed by the Sidak multiple comparisons test. A *p* value < 0.05 was considered to indicate statistical significance. *p < 0.05, **p < 0.01, ***p < 0.001, and ****p < 0.0001.

## Results

We designed a total of 5 sets of SNCA-shRNA sequence plasmids with GFP fluorescent tags, integrated them into lentiviruses, and transfected them into HEK293 cells to observe the degree of transfection. RT-qPCR experiments were performed. The results are shown in more detail in Additional file 1. To reduce experimental error, all animals received injections of lentiviruses including the gene of interest and an empty vector. None of the animals in the experiment died due to injection of the virus. To first ensure that the impact parameters were similar between groups, we examined the IH impactor output. The animals received contusions of the central thoracic spinal cord at T3, with an average contusion pressure of 402.26 ± 0.672 kilodynes, which was close to the initial contused peak force of 400 kilodynes. This difference was attributable to inertial compensation. In addition, 5 animals did not survive SCI, and 3 animals did not survive due to severe bladder dysfunction after surgery. Male animals were used because the majority of SCI cases occur in males [16].

### Temporal and spatial expression levels of α-Syn in the spinal cord after SCI

**Fig. 2:**
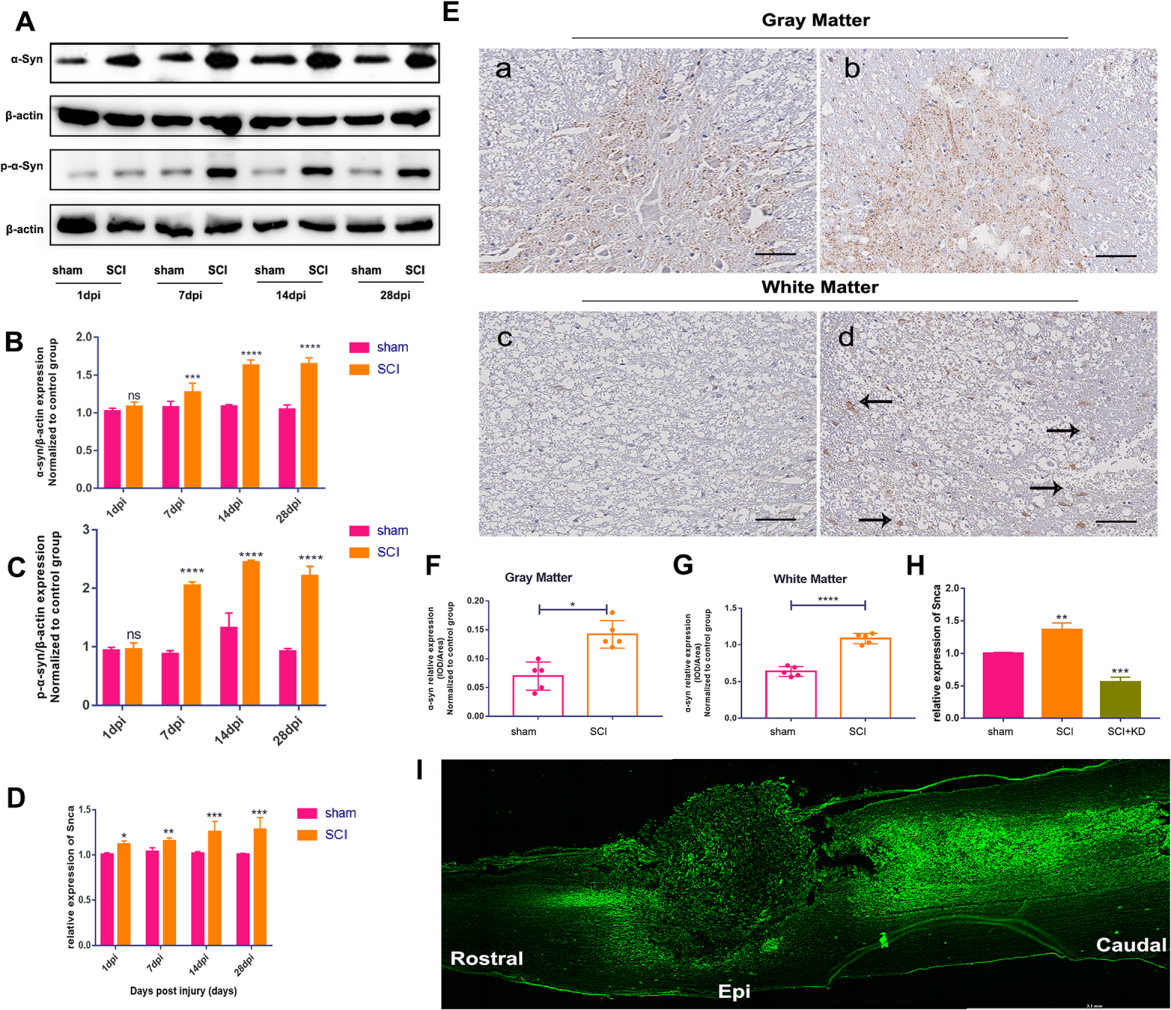
Expression and knockdown of α-Syn and SNCA in the spinal cord following SCI. **A** Representative Western blot results of α-Syn and p-α-Syn levels in the SCI group compared with the sham group at 1, 7, 14, and 28 days after SCI. **B**, **C** Quantification of the Western blot data in panel **A**, *n* = 5. **D** Relative expression levels of SNCA in the sham group and the SCI group over time, as detected by qRT-PCR, *n* = 5. **E** IHC was used to detect the expression of α-Syn in the GM and WM of the spinal cord on the 28th day after SCI. Scale bar = 100 μm. **F**, **G** Semi-quantitative analysis of the mean IOD values from IHC in panel **E**, *n* = 5. **H** Relative expression levels of SNCA in the three groups, as detected by qRT-PCR at 28 dpi, *n* = 5. **I** Distribution of lentivirus (LV; GFP+) expression in the spinal cord after SCI. Scale bar = 3 mm. Green fluorescence indicates a tissue transfected with LV. All data are presented as the mean ± SD. Comparisons between two groups were performed using Student’s *t* test or the Mann-Whitney test, as appropriate. One-way ANOVA and Tukey’s multiple comparisons test were used to analyse differences among groups

The oligomerization of α-Syn is a hallmark of chronic neurodegenerative synucleinopathies and causes its aggregation over time. In this study, we first examined the expression of α-Syn and phosphorylated α-Syn (p-α-Syn, Ser-129) in the injured site after SCI by Western blotting. The results showed that the expression of α-Syn at the early injury site at 1 dpi was higher in the SCI group than in the sham group, but the difference was not significant. The expression levels of α-Syn and p-α-Syn gradually increased from 7 dpi to 14 dpi and remained unchanged through 28 dpi in the SCI group relative to the sham group (Fig. 2a–c). RT-qPCR was used to detect transcriptional changes in the SNCA gene at the SCI site and indicated a gradual increase after SCI (Fig. 2d). Statistical analysis showed that, compared with that in the sham group, the transcriptional expression of SNCA increased after injury in the SCI group, and α-Syn and p-α-Syn protein levels increased significantly. There was a significant increase the first day after injury, and the most significant increase was observed on the 14th day.

Second, the expression of α-Syn in the GM and WM of the spinal cord was detected by IHC on the 28th day after SCI. Analysis of the mean IOD revealed that the expression of α-Syn in the WM was significantly higher in the SCI group than in the sham group (*p* < 0.0001) and showed a scattered punctate distribution (Fig. 2e, g). The GM expression was significantly higher in the SCI group than in the sham group (*p* = 0.023) (Fig. 2e, f).

To investigate the role of α-Syn in the spinal cord, we used a lentiviral vector to knock down the expression of SNCA in the injured area immediately after SCI. To confirm whether the lentivirus transduced the target tissue, we immediately injected a lentiviral GFP vector (LV-GFP; 10 μl; 1.0 × 10E9 TU/ml) into the injury sites of the rats. We examined spinal cord sections at 7 dpi and found that the lentivirus successfully transduced the spinal cord at the site of injury (Fig. 2i). Next, RT-qPCR was performed to verify the expression of SNCA in the sham group, SCI group, and SCI+KD group at 28 dpi. The SCI+KD group exhibited significantly lower expression than the SCI group, as shown in Fig. 2h.

### Blocking α-Syn improves neurological outcomes and functional recovery after SCI

**Fig. 3:**
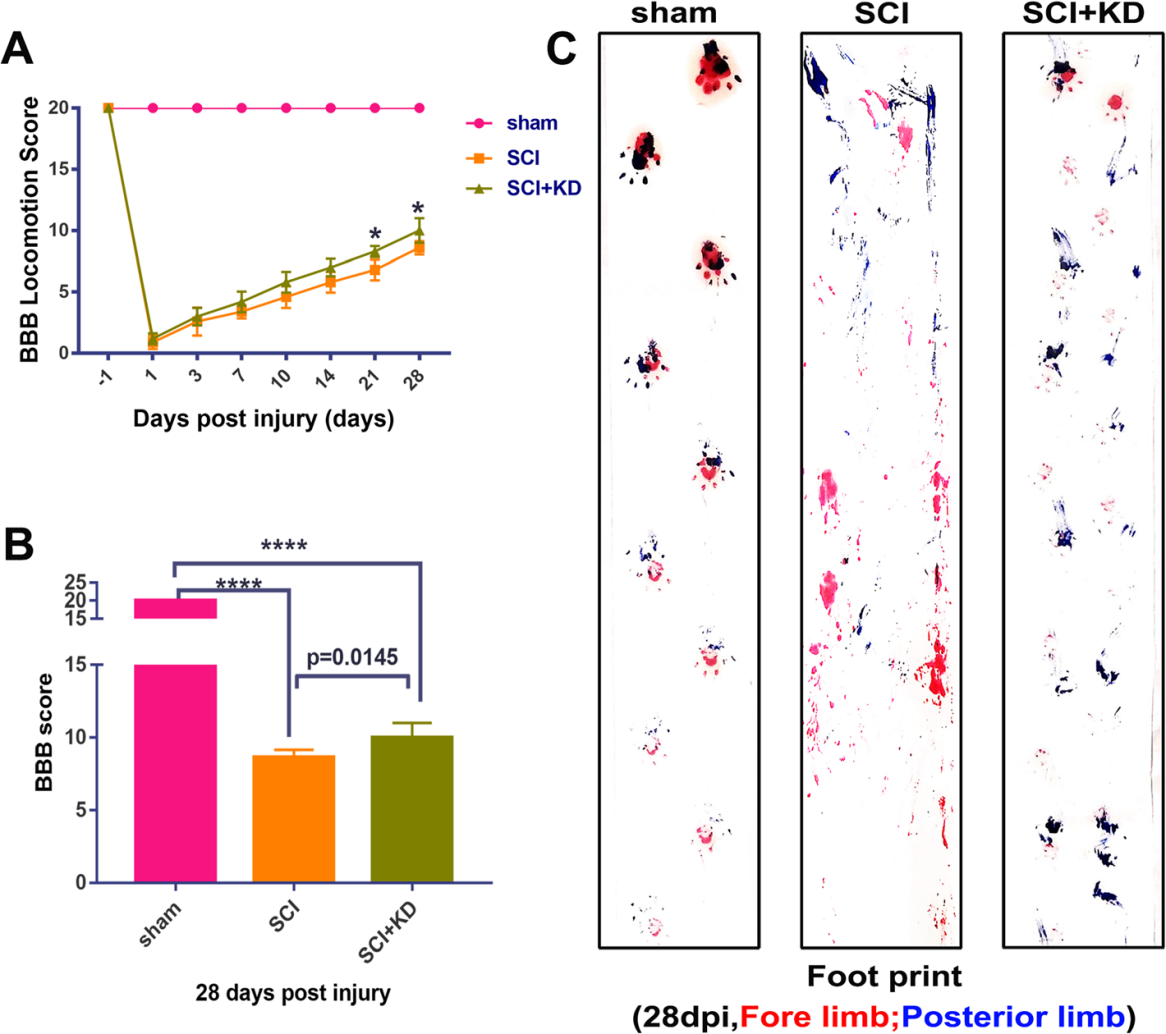
Lentivirus-mediated α-Syn knockdown improves neurological outcomes and functional recovery after SCI. **a** BBB exercise scale scores 1 day before SCI and 1, 3, 7, 10, 14, 21, and 28 days after SCI in the sham, SCI, and SCI+KD groups (*n* = 5). **b** Quantification of the BBB exercise rating scale scores on day 28 in each group, *n* = 5. **c** Results of the footprint analysis experiments for the different groups. All data are presented as the mean ± SD. The BBB motor function scores were analysed by two-way repeated-measures ANOVA followed by the Sidak multiple comparisons test

We next assessed the therapeutic effect of α-Syn blockade after SCI. We used BBB motor function scores and footprint analysis to assess motor function for 28 days after SCI. Normal motor function was scored as 21 points. The SCI and SCI+KD groups exhibited scores significantly lower than those in the sham group (*p* < 0.0001). As shown by the BBB exercise scale scores, no significant differences were found between the SCI group and the SCI+KD group early after SCI (1, 3, 7, 10, and 14 dpi); the SCI+KD group scores were comparable with the SCI group scores (*p* = 0.99 (1 dpi), *p* = 0.97 (3 dpi), *p* = 0.49 (7 dpi), *p* = 0.07 (10 dpi), and *p* = 0.07 (14 dpi)). As the observations continued, we noted better functional recovery in the SCI+KD group than in the SCI group, as indicated by better scores *p* = 0.01 (21 dpi) and *p* = 0.02 (28 dpi)) (Fig. 3a). Figure 3b shows the significant difference in the recovery of motor function at the end of observation on day 28.

In the footprint analysis, the LV-SNCA-shRNA-treated SCI rats showed relatively consistent hindlimb coordination (blue ink) 28 days after SCI compared with the empty vector-treated SCI rats. The SCI+KD group exhibited a gait more similar to that of the sham group. In contrast, SCI animals receiving empty vector treatment showed incongruous gaits and extensive drag of both hindlimbs, as indicated by ink streaks (Fig. 3c). These data indicate that downregulation of α-Syn after SCI improves motor function in SCI rats.

### Downregulation of α-Syn after SCI can ameliorate pathological findings in injured tissue

**Fig. 4:**
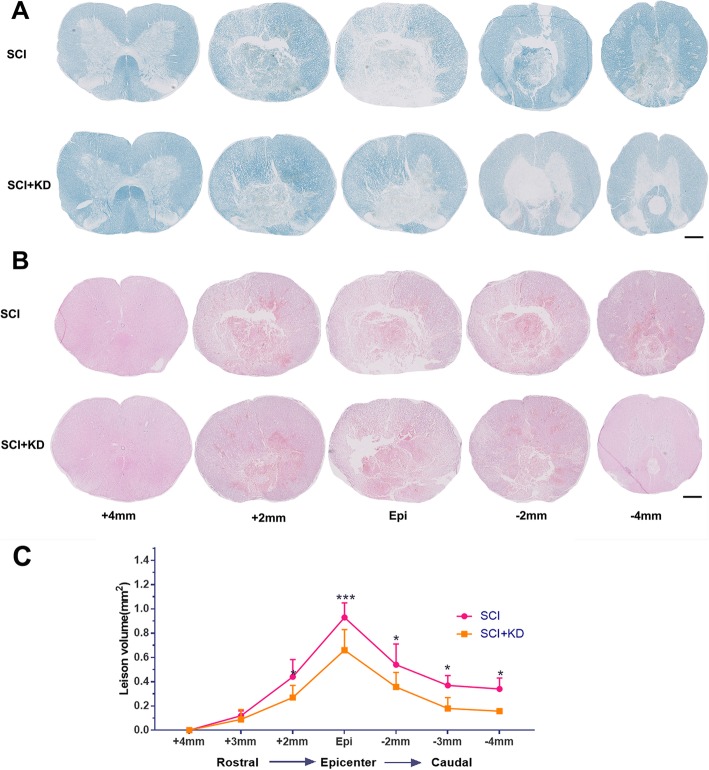
Downregulation of α-Syn after SCI can ameliorate pathological findings in injured spinal cord tissue. **a** Representative images showing LFB staining after SCI. Scale bar = 1 mm. **b** Representative images showing HE staining after SCI. Scale bar = 1 mm. **c** Quantitative analysis of the results in panel **a**. All data are presented as the mean ± SD, *n* = 5. For staining that was performed 2 mm from both ends in the SCI+KD group and the SCI group, *p* < 0.05; for staining that was performed at the centre of the SCI area in the SCI+KD group, *p* < 0.001 compared with that in the SCI group. One-way ANOVA and Tukey’s multiple comparisons test were used to analyse differences among groups

We examined the expression of GFAP and Iba1 around the injury site on the first post-operative day by IHC. There was no significant difference between the SCI group and the SCI+KD group; however, we found that compared with the sham group, the other two groups exhibited significant increases in glial cell numbers and activity, very obvious changes in microglial morphology, retraction of cell protrusions, and obvious enlargement and thickening/clustering of the cell body (see Additional file 2: Figure S3). In summary, glial cell activation immediately after injury may be associated with preformed α-Syn. We also assessed the extent of tissue preservation 7 days after SCI. We performed morphometric analyses of LFB and HE staining of paraffin-embedded spinal cord tissue and made serial sections of the tissue 4 mm away from the centre of the injury. Cross-sections of tissues taken from areas different distances from the injury site (2 to 4 mm from the centre of the lesion in the rostral-caudal plane) were quantified. LFB staining was used to observe the loss of myelin in sections from the middles of the two ends of the spinal cord (Fig. 4a,c). In addition, we performed HE staining to observe the infiltration of blood cells (dark red cells) in sections from the middles of the two ends of the spinal cord and the surrounding lesions (Fig. 4b). The staining objectively showed that infiltration of large amounts of peripheral blood above and below the level of the SCI, indicating destruction of the BCSB. Compared with the SCI+KD group, the SCI group showed a greater amount of peripheral blood infiltration, suggesting that the BCSB functional damage was more severe.

At the distal end of the injured spinal cord, there was a large demyelinated cavity near the central canal, and there were large and small demyelinated lesions in the WM accompanied by peripheral blood cell infiltration, which are pathological features of secondary injury. In addition, the SCI+KD group exhibited reduced myelin loss and peripheral blood cell infiltration in the distal end of the spinal cord.

### Reduced α-Syn levels can reduce microglial numbers and activation

**Fig. 5:**
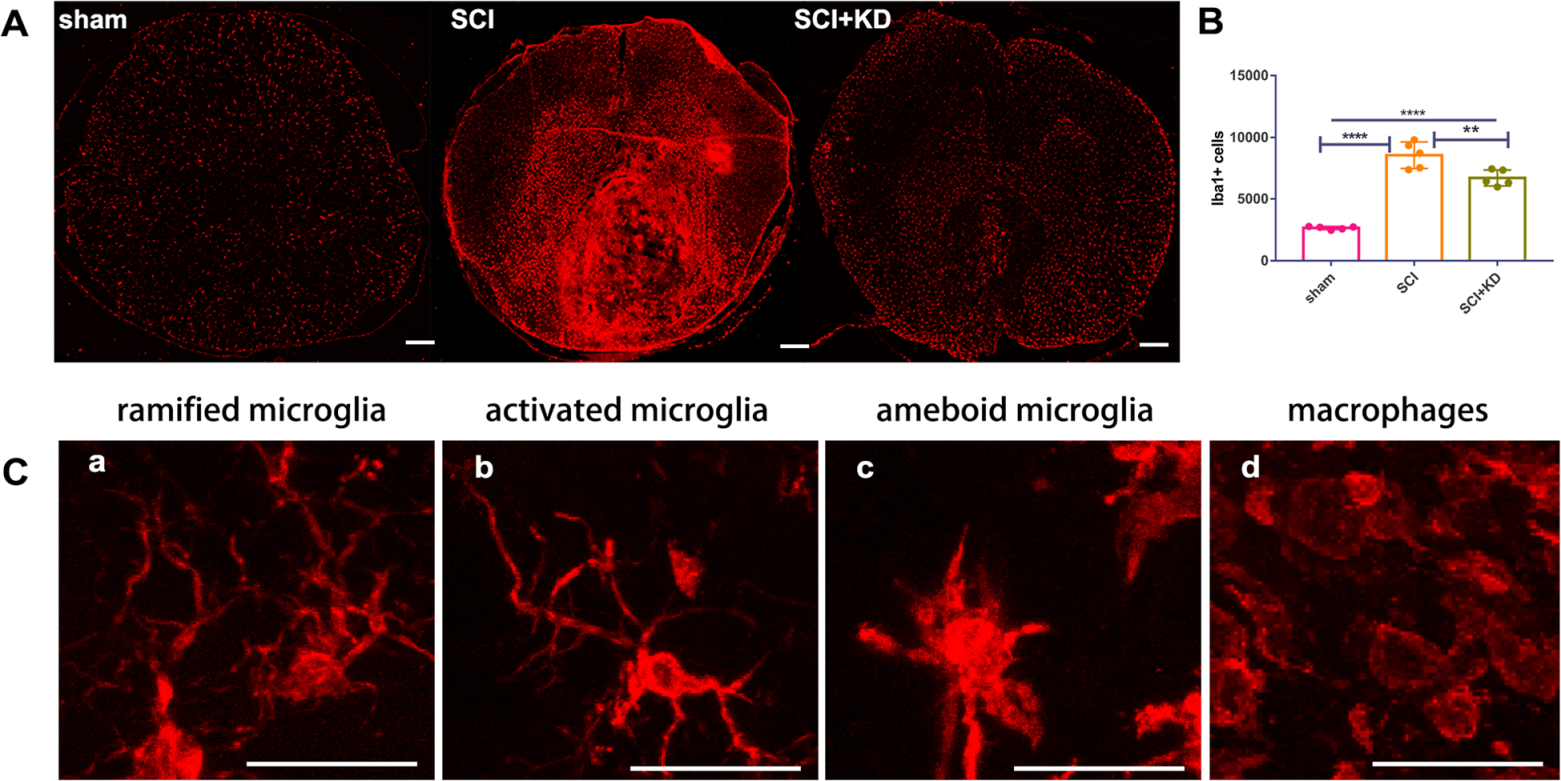
Reduced levels of α-Syn can reduce the microglial numbers and activation. **A** Representative images of Iba1 IF staining after SCI. Scale bar = 1 mm. **B** Quantitative analysis of the results in panel **A**. All data are presented as the mean ± SD, *n* = 5. One-way ANOVA and Tukey’s multiple comparisons test were used to analyse differences among groups. The *p* values for the SCI+KD and SCI groups compared with the sham group were *p* < 0.05 and *p* < 0.001, respectively. **C** Under high magnification, four different typical forms of Iba1+ cells were observed

Microglia and astrocytes are the main effectors of neuroinflammation after injury [17, 18]. To analyse these cells, we performed IF staining of microglia (Iba1) in frozen sections on the 28th day after SCI (Fig. 5a). Panoramic scanning of tissue sections was performed under a × 5 objective, and the numbers of microglia before and after injury were compared. The number of Iba1+ cells in the SCI group was significantly higher than that in the SCI+KD group, and the numbers in both groups were significantly higher than that in the sham group (Fig. 5b). High-power microscopy showed that the sham group mainly contained a small number of highly branched microglia (Fig. 5c). The SCI group and the SCI+KD group showed considerable branch retraction, thickening, amoeba-like morphology, and peripheral blood infiltration of macrophages. The SCI+KD group showed less microglial activation and macrophage infiltration than the SCI group.

### Reductions in α-Syn promote M1-to-M2 phenotypic polarization of microglia/macrophages

**Fig. 6:**
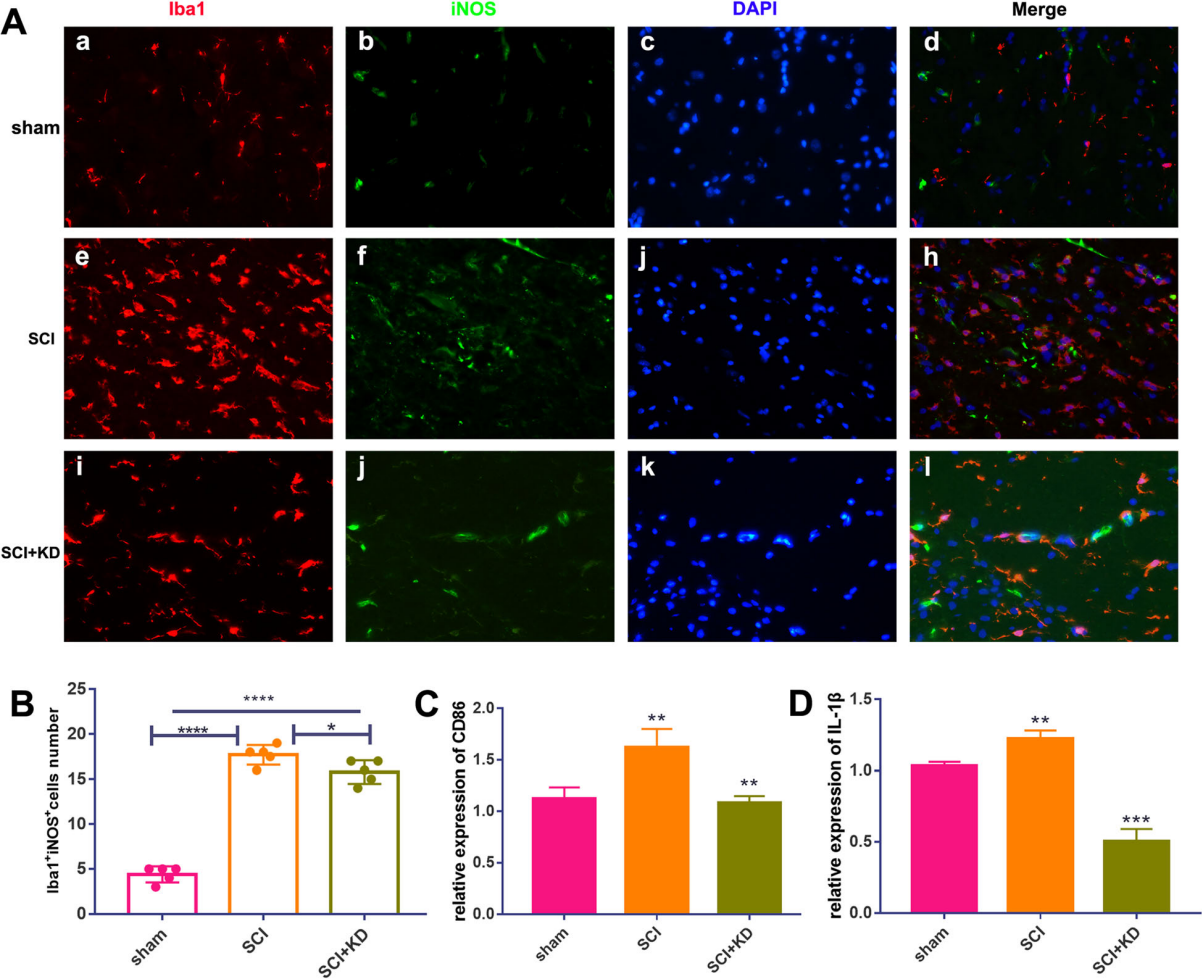
Reduced α-Syn levels significantly decrease iNOS production in microglia/macrophages. **A** Representative images showing Iba1+/iNOS+ IF staining after SCI. Scale bar = 100 μm. **B** Quantitative analysis of the results in panel **A**, *n* = 5. **C**, **D** Relative mRNA expression levels of CD86 and IL-1β in all groups, as detected by qRT-PCR. All data are presented as the mean ± SD, *n* = 5. One-way ANOVA and Tukey’s multiple comparisons test were used to analyse differences among groups

**Fig. 7:**
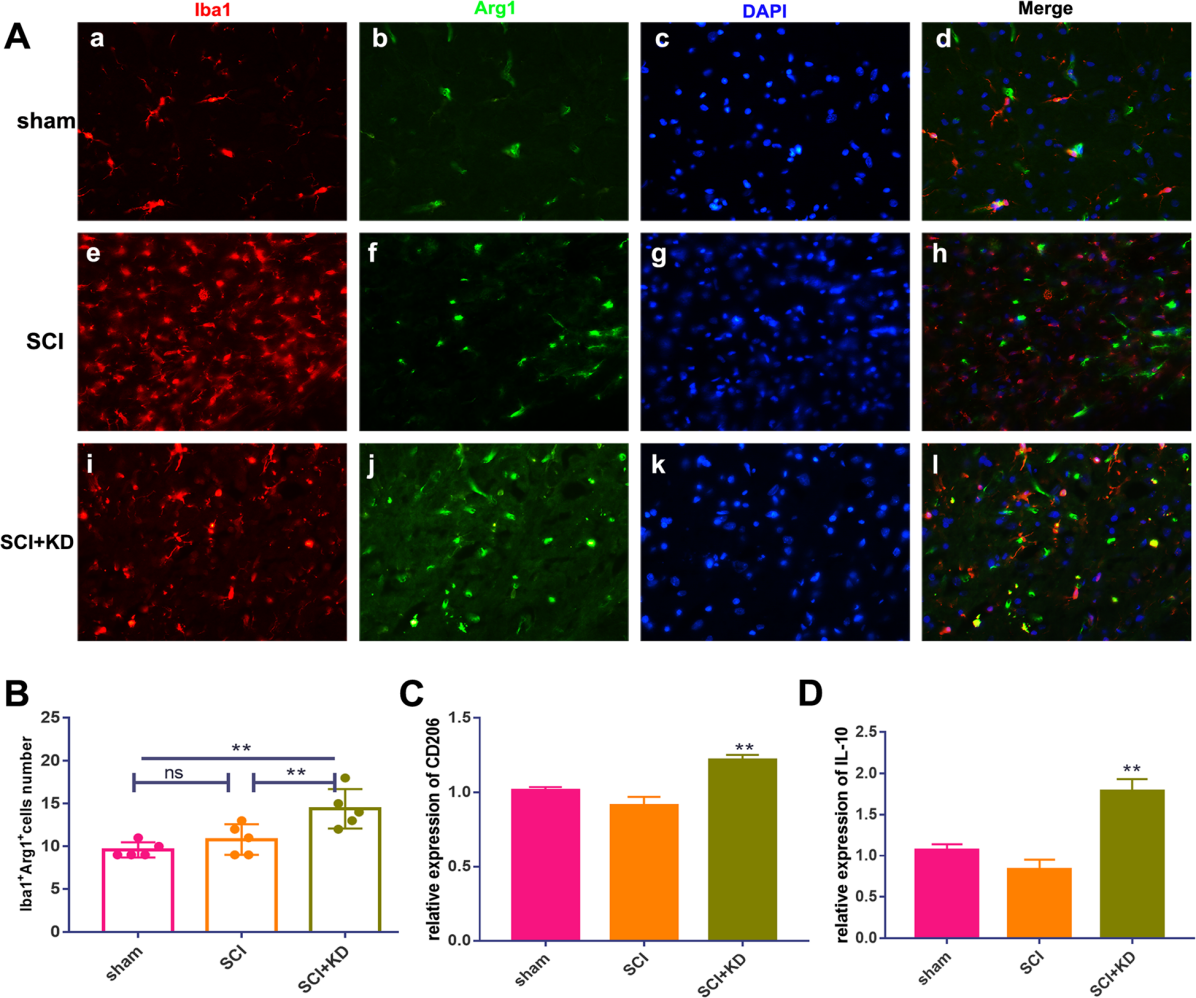
Reduced α-Syn levels significantly increase Arg1 expression in microglia/macrophages. **A** Representative images showing Iba1+/Arg1+ IF staining after SCI (a, e, and i show microglia (Iba1); b, f, and j show iNOS; and c, j, and k show cell nuclei (DAPI)). Scale bar = 100 μm. **B** Quantitative analysis of the results in panel **B**, *n* = 5. **C**, **D** Relative mRNA expression levels of CD206 and IL-10 in all groups, as detected by qRT-PCR. All data are presented as the mean ± SD, *n* = 5. One-way ANOVA and Tukey’s multiple comparisons test were used to analyse differences among groups

Activated M1-type microglia release NO after traumatic and ischaemic injury to the CNS, and this initiates oxidative stress and a neurotoxic cascade in neurons. In addition, in M2-type microglia, arginase1 (Arg1) is known to block uncoupling of nitric oxide synthase (NOS) [19]. We observed Iba1+/iNOS+ double-labelled microglia by IF at 28 dpi. The number of these double-labelled microglia was significantly lower in the SCI+KD group than in the SCI group but was still higher in the SCI+KD group than in the sham group (Fig. 6a, b). The number of Iba1+/Arg1+ double-labelled cells was significantly higher in the SCI+KD group than in the SCI group and the sham group (Fig. 7a, b). This finding indicates that after SCI, reductions in α-Syn contribute to increased expression of Iba1+/Arg1+ M2-type cells, decrease the expression of Iba1+/iNOS+ M1-type cells, and promote the polarization of M1 microglia/macrophages to the M2 phenotype.

In addition, using RT-qPCR, we further tested transcriptional levels in M1/M2 microglia/macrophages in the injury site after knocking down α-Syn expression after SCI. The relative mRNA expression levels of CD86, CD206, and the cytokines IL-1β and IL-10 were detected in the injured tissues on day 28, and the changes in the pro-inflammatory (M1) response and anti-inflammatory (M2) response at the transcriptional level were observed. Among these changes, the expression of the pro-inflammatory cytokine IL-1β and the M1-type microglial/macrophage protein CD86 in the SCI+KD group was significantly decreased (Fig. 6c, d). In contrast, the expression of the anti-inflammatory cytokine IL-10 and the M2-type microglial/macrophage protein CD206 in the SCI+KD group was elevated (Fig. 7c, d). All of the above results show that reductions in α-Syn promote the polarization of M1 microglia/macrophages to the M2 phenotype and promote the pro-inflammatory response over the anti-inflammatory response.

### Silencing α-Syn improves the immune microenvironment after SCI

**Fig. 8:**
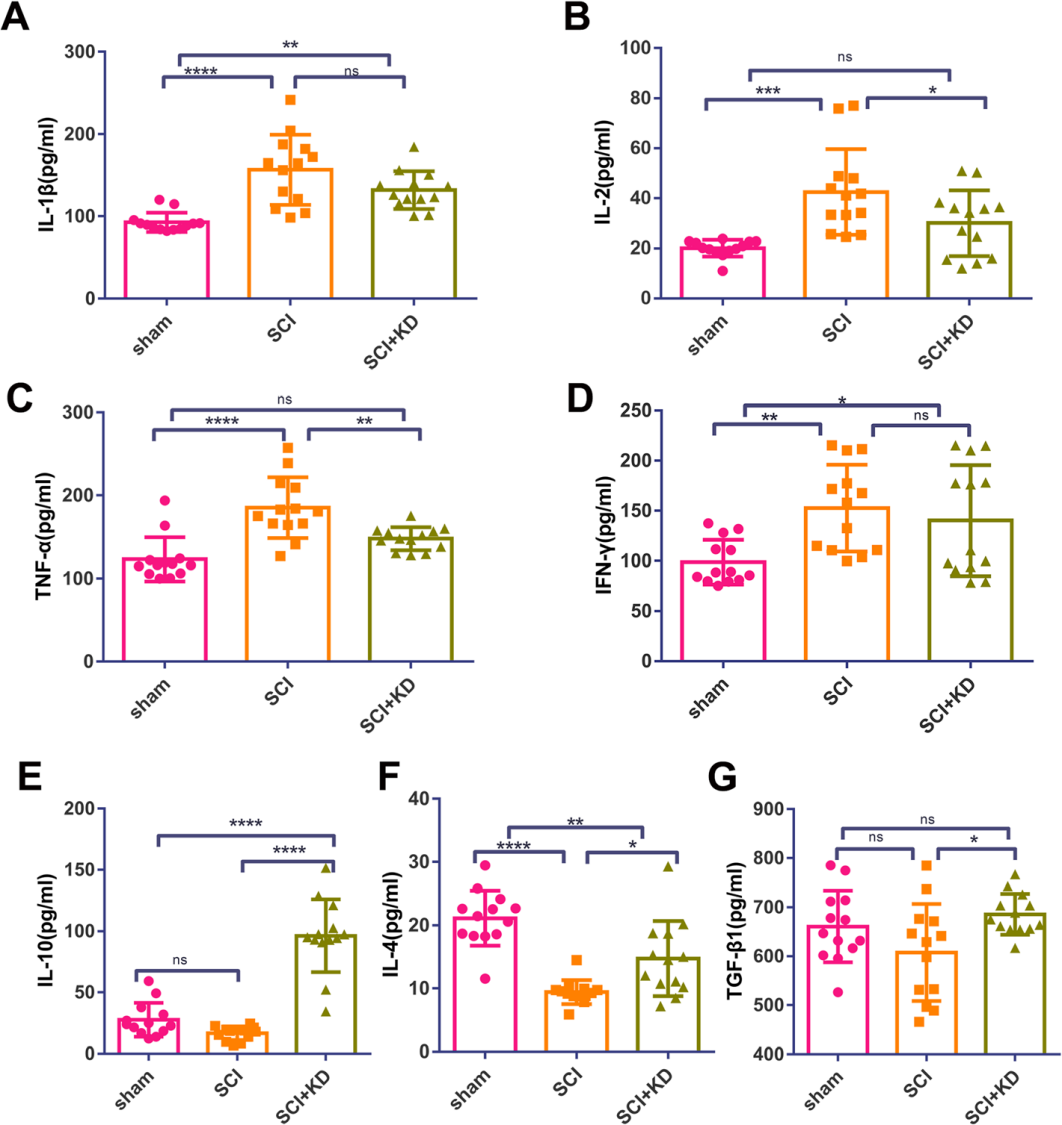
Knockdown of α-Syn promotes changes in peripheral serum inflammation after SCI. **a**–**d** Changes in the levels of the pro-inflammatory cytokines IL-1β, IL-2, TNF-α, and IFN-γ, *n* = 13. **e**–**g** Changes in the levels of the anti-inflammatory cytokines IL-10, IL-4, and TGF-β1, *n* = 13. All data are presented as the mean ± SD. One-way ANOVA and Tukey’s multiple comparisons test were used to analyse differences among groups

The early recruitment of microglia/macrophages after SCI is characterized by the release of pro-inflammatory cytokines that cause cell death and tissue degeneration following injury [20]. ELISA was used to analyse the expression of the pro-inflammatory cytokines TNF-α, IL-1β, IL-2, and IFN-γ (Fig. 8a–d) and the anti-inflammatory cytokines IL-10, IL-4, and TGF-β1 in the serum of each group on the 28th day after the operation. The levels of the anti-inflammatory cytokines IL-10, IL-4, and TGF-β1 in the SCI+KD group were higher than those in the SCI group, and the expression of IL-10 was significantly higher than that in the SCI group (Fig. 8e–g). The results show that targeting the knockdown of α-Syn promotes the transformation of peripheral blood from a pro-inflammatory to an anti-inflammatory state after SCI, thereby improving the immune microenvironment to allow neuronal survival and reduce secondary damage.

### Decreases in α-Syn can reduce the activation of microglia/astrocytes in the central canal of the spinal cord after SCI

In the above experiment, considerable peripheral blood cell infiltration and demyelination in the cavity around the central canal of the spinal cord were observed after SCI. Therefore, we next evaluated the effect of reducing α-Syn on microglial/astrocyte proliferation in the central spinal canal 28 days after SCI. It is currently believed that astrocytes exist in three states, namely, a resting state, an activated state, and a proliferative state. Reactive astrocyte proliferation is mainly identified by increased expression of GFAP [21].

**Fig. 9:**
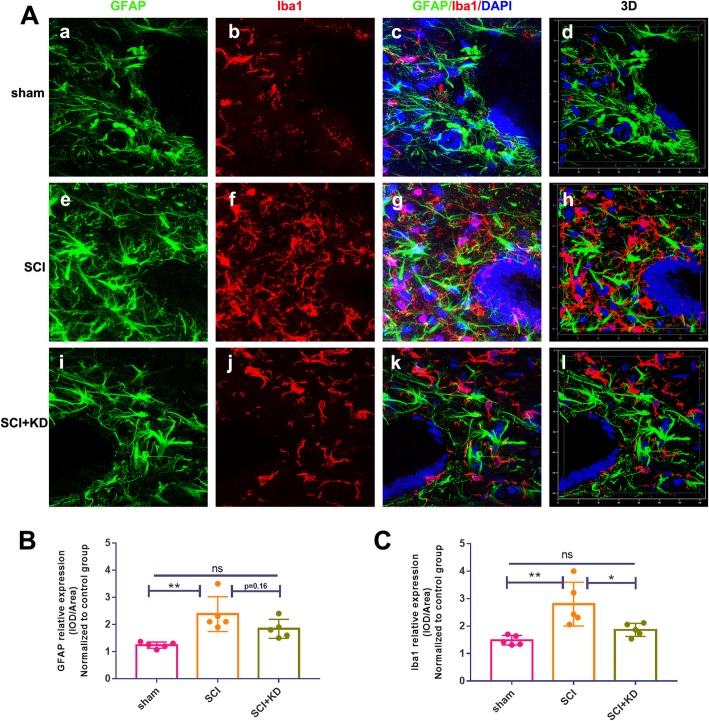
Reductions in α-Syn can reduce microglial/astrocyte activation in injured spinal cord tissue. **A** Representative images showing GFAP+/Iba1+ double staining after SCI by IF (a, e, and i show microglia (Iba1); b, f, and j show microglia (Iba1); c, j, and k show nuclei (DAPI); and d, h, and i show the 3D mode). Scale bar = 50 μm. **B** Quantitative analysis of the mean fluorescence intensity of GFAP in panel **A**. **C** Quantitative analysis of the mean fluorescence intensity of Iba1 in panel **A**. All data are presented as the mean ± SD, *n* = 5. One-way ANOVA and Tukey’s multiple comparisons test were used to analyse differences among groups

Evaluation of GFAP+/Iba1+ double staining in SCI lesions showed that the astrocytes in the SCI group and the SCI+KD group were hypertrophied, and their branches were thickened, showing morphological changes associated with reactive astrocytes. Compared with that in the SCI group, the average fluorescence intensity of GFAP in the SCI+KD group was downregulated at 28 days, but the difference was not significant (*p* = 0.16). The GFAP signals remained elevated (1- to 1.5-fold) in the SCI+KD group compared with the sham group (*p* < 0.05, *n* = 5/group) (Fig. 9a, b). Consistent with our above experimental data, knockdown of α-Syn, compared with empty vector treatment, significantly attenuated microglial signals in the spinal cord central canal at 28 dpi (*p* < 0.05, *n* = 5/group) (Fig. 9a, c). Importantly, we observed tight junctions between microglia and astrocyte branches through 3D image visualization, and the SCI group showed closer connections than the other groups. These findings indicate that α-Syn knockdown can preferentially reduce microglial activation, reduce astrocytic density, and further reduce reactive astrocyte activation and neuroinflammation near the central canal of the spinal cord.

### Knocking down α-Syn can reduce the expression of MMP-9 in the central canal of the spinal cord

**Fig. 10:**
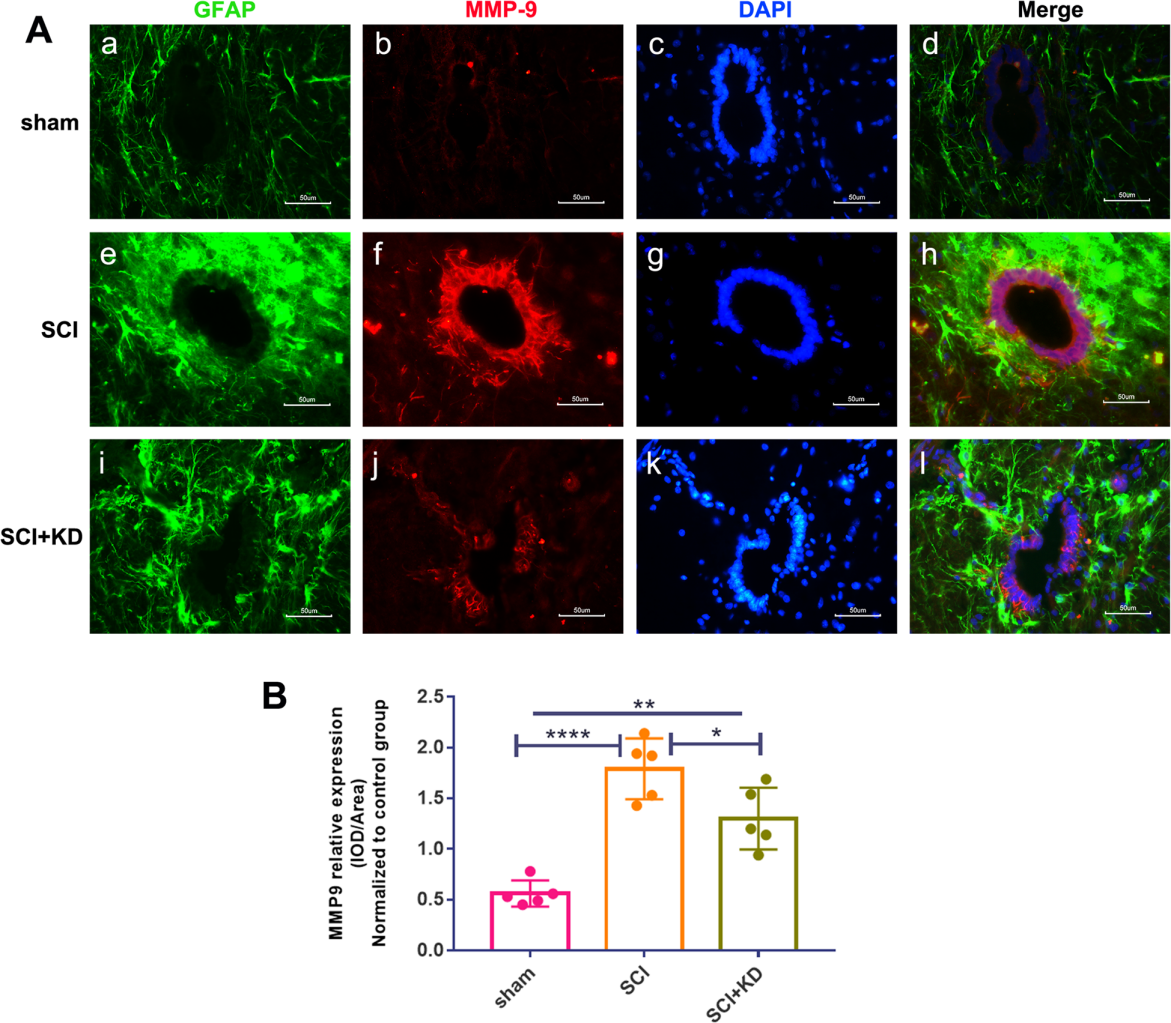
α-Syn can reduce the expression of MMP-9 in the central canal of the spinal cord. **A** Representative images showing GFAP+/MMP-9+ double staining by IF after SCI. Scale bar = 50 μm. **B** Quantitative analysis of the mean fluorescence intensity of MMP-9 in panel **A**. All data are presented as the mean ± SD, *n* = 5. One-way ANOVA and Tukey’s multiple comparisons test were used to analyse differences among groups

Since we determined that reducing α-Syn attenuates pro-inflammatory mediator levels and neuroinflammatory responses, we next examined whether this change inhibits the activity of matrix metalloproteinases (MMPs) after SCI. MMP-9 plays a major role in tissue degeneration and reconstruction after injury [22, 23]. GFAP+/MMP-9+ double staining was analysed by IF. Compared with that in the SCI group, the expression of MMP-9 in injured spinal cord tissue in the SCI+KD group was decreased on the 28th day after injury (*p* < 0.05, *n* = 5/group) (Fig. 8a, b). Studies have shown that MMP-9 and astrocytes are involved in the homeostasis and maintenance of the BCSB and that elevated MMP-9 expression leads to endothelial cell dysfunction [24]. Our results indicate that knockdown of α-Syn after SCI can reduce MMP-9 expression in the central canal of the spinal cord (Fig.10A—b, f, j). As shown in Fig. 10A—d, h, i, MMP-9 was expressed along the endothelial cells of the spinal cord, and astrocytes were distributed around the MMP-9-expressing cells. Increased expression of MMP-9 enhances the permeability of the BCSB, promotes the infiltration of peripheral inflammatory cells, and aggravates the development of neuroinflammation after SCI. Knocking down α-Syn expression contributes to reductions in MMP-9, thereby reducing peripheral blood infiltration and inflammation.

### Hypothesis of the role of α-Syn in neuroinflammation after SCI

**Fig. 11:**
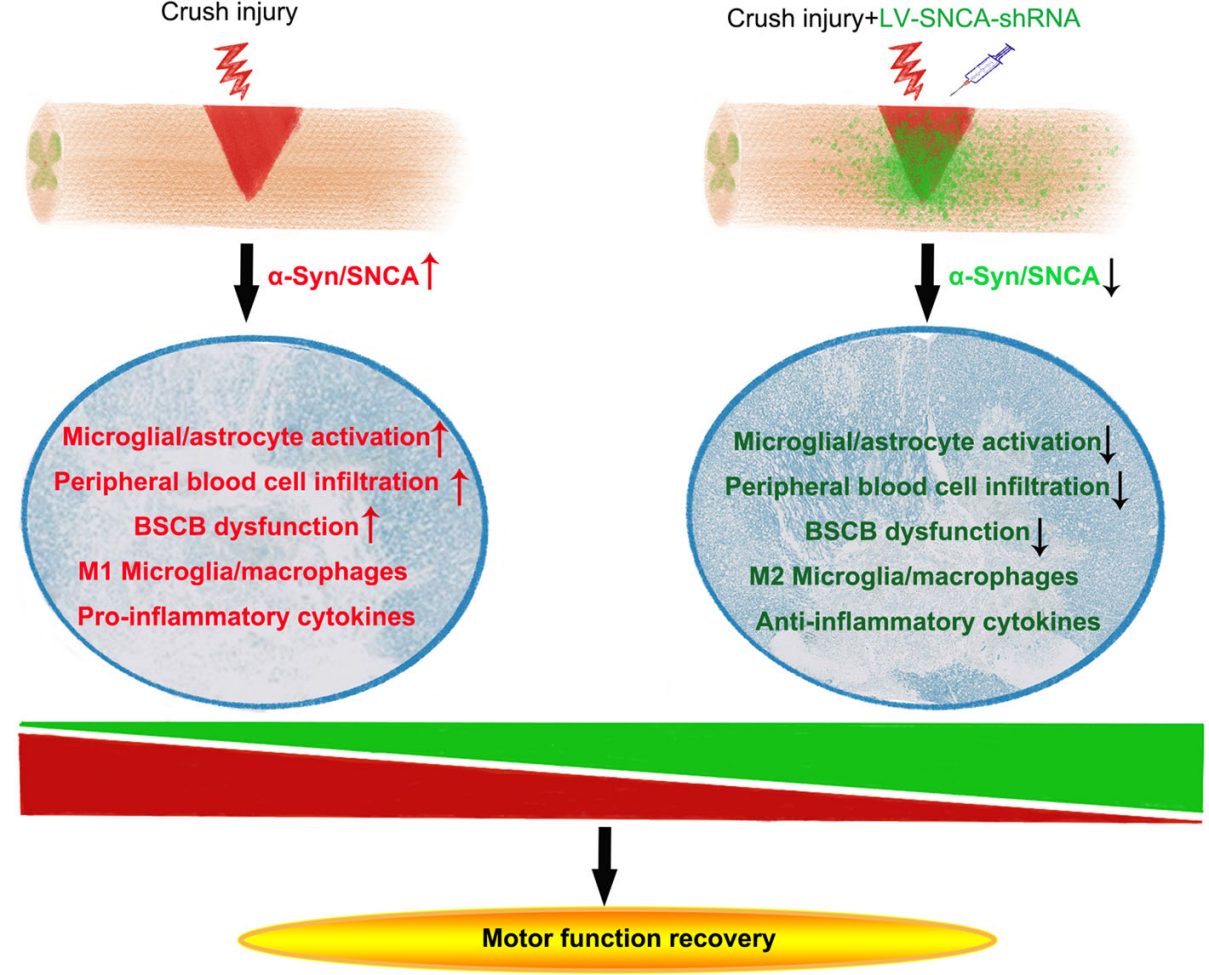
Schematic diagram of the role of α-Syn in neuroinflammation after SCI. BSCB, blood-spinal cord barrier

We observed increased α-Syn expression, microglial/astrocyte activation, peripheral blood cell infiltration, severe BCSB dysfunction, and M1-type microglial/macrophage transformation accompanied by considerable pro-inflammatory cytokine release secondary to SCI, which resulted in a severe loss of distal myelin at the proximal end of the injured area. We knocked down α-Syn expression with LV-SNCA-shRNA, which ameliorated the dysfunction and pathology, reduced CNS inflammation, and promoted anti-inflammatory responses. Furthermore, α-Syn knockdown improved BCSB function and restored the immune microenvironment, which conferred neuroprotection after SCI, as shown in Fig. 11.

## Discussion

We generated a Sprague-Dawley rat model of spinal cord contusion injury of the T3 segment. Injuries to high-level segments are clinically more common than injuries to lower segments, and compared with other models, contusion of T3 is safer with a higher survival rate [25]. Secondary SCI lesions develop within seconds to months after the primary phase and have been the focus of a series of modern medical interventions. In the present study, we showed that the expression of α-Syn and p-α-Syn (Ser129) was significantly increased in the early stage of SCI. In addition, the increased expression was mainly concentrated in the WM of the spinal cord with a scattered distribution. LV-SNCA-shRNA transfection knocked down spinal α-Syn early after SCI. We found that α-Syn is an important promoter of neuroinflammatory factors. During secondary injury after SCI, α-Syn reduced the activation of microglia/astrocytes and the expression of iNOS and promoted the transformation of M1 macrophages to the M2 phenotype, and these changes were accompanied by increases in Arg1 and IL-10 expression. Significant increases in the expression of these cytokines regulate neuroinflammation in the spinal cord. The SCI+KD group also showed decreases in CD86+ M1-type microglia and IL-1β levels and an elevated CD206+ M2 phenotype at the transcriptional level. The peripheral blood serum levels of IL-1β, TNF-α, and IL-2 were also decreased, and the levels of the anti-inflammatory cytokine IL-10 were significantly increased. In addition, we found that the SCI+KD group exhibited reduced expression of MMP-9, which may have improved the function of the BCSB, a critical barrier for central immunity and peripheral immune stabilization.

The α-Syn protein encoded by the SNCA gene is a small (14-kD) presynaptic nerve terminal protein [26–28]. Intracellular aggregation of this 140-amino-acid neuronal protein is thought to play an important role in the pathogenesis of neurodegenerative diseases, including Parkinson’s disease (PD) and Alzheimer’s disease (AD) [29, 30]. Over the last decade, genome-wide association studies (GWASs) and candidate gene-based approaches have implicated SNCA mutant-type as a highly significant genetic risk factor for synucleinopathies [55]. A study by Sakurai et al. investigated biomarkers of SCI, specifically DJ-1, PINK1, and α-Syn, in rabbits. After transient spinal cord ischaemia, α-Syn participates in spinal motor neuron death, possibly through oxidative stress [31]. Another study by Busch et al. revealed that SNCA accumulation is prioritized and is correlated closely with an increased risk of neuronal death after SCI [32]. A study by Kim et al. showed that transient focal cerebral ischaemia upregulates α-Syn protein expression and nuclear translocation in adult rodent brain neurons and that knockdown of α-Syn reduces cerebral infarct area, mitochondrial rupture, oxidative stress, apoptosis, and autophagy and promotes better neurological recovery after cerebral ischaemia [33]. These results indicate that abnormal aggregation of α-Syn promotes neuronal death not only in the contexts of chronic neurodegenerative diseases such as PD but also in ischaemic lesions. In another recent study, miR-7 was shown to attenuate α-Syn-mediated secondary brain injury after stroke, possibly by acting on multiple downstream targets of apoptosis, autophagy, oxidative stress, and mitochondrial damage, and to prevent neuropathology associated with stroke [34]. In addition, various animal studies have identified the ability of α-Syn to induce apoptotic gene expression, thereby promoting neuronal death in vivo [35, 36]. Notably, Feng et al. showed that downregulation of SNCA promotes ciliary neurotrophic factor (CNTF) expression, inhibits neuronal apoptosis, and promotes nerve regeneration [9]. In addition to in vitro and animal models, patients with CNS injury also exhibit elevated levels of α-Syn in the cerebrospinal fluid and axons [37–40]. Population-based studies have also shown increased incidences of neurodegenerative diseases (including PD and AD) in patients with previous SCIs [41, 42].

Abnormal deposition of proteins, including amyloid-β, α-Syn, and prions, wshich can easily cause neuroinflammation, not only directly occurs in neurons but also involves glial cells [43]. α-Syn is involved in neuroinflammation and subsequent apoptosis in the CNS. However, to date, only a few studies (primarily in vivo studies) have investigated the pathophysiological effects of α-Syn on affected nerve tissue after SCI. A recent study by Qiao et al. found a role for α-Syn (SNCA) in microglial migration [12]. Another study by this team isolated primary microglia from Sprague-Dawley rats and exogenously exposed them to three different doses of α-Syn oligomers, finding that α-Syn stimulates toll-like receptor 2 (TLR2) by inducing microglial activation after SCI [44]. These findings indicate that α-Syn is an important inducer of neuroinflammation and concurrent neuronal loss after SCI. Consistent with our experimental results, it has been determined that α-Syn is closely related to neuroinflammation after SCI. Microglia can exhibit an M1 or M2 phenotype based on pro-inflammatory or anti-inflammatory properties, respectively. Microglia that produce high levels of the pro-inflammatory factors iNOS, IL-2, IFN-γ, TNFα, IL-1β, and IL-12 are generally considered to be pro-inflammatory (M1-type). On the other hand, microglia that show increased levels of Arg1, CD206/Mrc1, Fizz1, Ym1, and IL-10 exhibit more of an anti-inflammatory (M2-type) phenotype [45]. A study on a PD model showed that overexpression of neuronal α-Syn results in increases in NADPH oxidase activity, OX-6+ microglial cell numbers, and phagocytic activity marker (CD68) levels. Inflammatory M1 microglial/macrophage phenotype markers, such as iNOS, TNF-α, IL-1β, and IL-6, are also elevated [46]. In our study, knockdown of α-Syn promoted the conversion of M1 microglia/macrophages to the M2 phenotype, and this effect was accompanied by increased expression of Arg1. Further research is needed to elucidate the factors that mediate microglia or peripheral macrophage activity in the context of SCI and to determine the exact mechanisms of action of these cells.

Another report indicated that in PD, α-Syn induces peripheral congenital immunity followed by MHC-II adaptive immunity and changes in T-helper lymphocyte subsets [47]. Elevated neuronal α-Syn levels promote microglial activation following spinal cord ischaemia/reperfusion injury, a process that is mediated by TLR2, but not TLR3 or TLR4, cell activation and activation of the p38 MAPK and NF-κB pathways [48].

Reactive astrocytes that express GFAP are important effectors of neuroinflammation. We detected increased numbers of astrocytes around the central canal in the surviving tissue at the injured site after SCI, although no significant differences were detected. However, we found that astrocytes were hypertrophic after injury, which is characteristic of activated astrocytes. Under acute injury stress, astrocytes first change their cell morphology and can then release pro-inflammatory factors such as cytokines, prostaglandins, neurotrophic factors, and neuromodulators (e.g. ATP and NO), which may indicate enhancement of defence capabilities. However, most studies have shown that, with long-term sustained activation, reactive astrocytes eventually transform into glial scars that impede axonal growth in the chronic phase [49].

The highly precise function of the BCSB allows endothelial cells to tightly regulate the stability of the CNS. This structure plays a key role in maintaining the normal functions of neurons and in protecting the CNS from toxins, pathogens, inflammatory factors, and diseases. Destruction of the BCSB is an important pathophysiological basis for secondary injury after SCI. Increased MMP-9 activity is associated with several pathologies in the context SCI, including enhanced astrocyte migration, glial scar formation [50], and the destruction of the BCSB. MMP-9-deficient mice are less sensitive to BCSB destruction than wild-type mice, and this decrease in sensitivity reduces neutrophil infiltration and functional recovery after SCI [51]. We found that rats subjected to SCI and transfected with LV-SNCA-shRNA exhibited reduced MMP-9 expression in the central canal of the spinal cord and reduced spinal cord haemorrhage, as evidenced by HE staining. It has been shown that the reduced neuroprotective effect of α-Syn in the spinal cord is at least partly due to inhibition of BCSB destruction and haemorrhage after SCI. Interestingly, there is increasing evidence that α-Syn is produced not only in the CNS but also in the periphery. Several studies have shown that there are multiple pathways of α-Syn transmission from the periphery to the brain, and α-Syn levels in the plasma are approximately 10 times higher than those in the cerebrospinal fluid (CSF) [52, 53]. Typically, α-Syn transport in the blood is controlled by the blood-brain barrier (a dynamic barrier between the peripheral circulatory system and the CNS). Free α-Syn protein is transported bidirectionally (from the blood to the brain and in the reverse direction) through this barrier [54]. Based on our data and those from others, we hypothesize that after SCI, reducing α-Syn at the SCI site reduces neuroinflammation, ameliorates BCSB dysfunction, and prevents excessive free α-Syn in the blood from being transported to the CNS, further improving the homeostatic environment after SCI.

Although LV-SNCA-shRNA was injected early, the in vivo transfection took 4–5 days, so we were unable to observe neuroinflammatory changes in the acute phase in the LV-SNCA-shRNA treatment group. After SCI, considerable infiltration of peripheral monocytes occurred due to the destruction of the BCSB, and we could not determine the impact of these processes on inflammation. Specific cell markers should be detected in subsequent studies. We demonstrated that LV-SNCA-shRNA treatment improves neuroinflammation and promotes functional recovery after SCI by promoting the M1/M2 phenotype. However, the exact signalling pathway is not described here, and the relationship between inflammation and synucleinopathies is unclear.

In summary, the data reported here demonstrate that the balance between pro-inflammatory and anti-inflammatory microglial/macrophage phenotypes affects the overall inflammatory state following SCI and is also the main factor controlling the survival of neurons.

## Conclusion

Lentivirus-mediated downregulation of α-synuclein reduces neuroinflammation and improves functional recovery in rats with SCI. We tested α-Syn activation and microglial phenotypes, and we demonstrated that reducing α-Syn levels promotes the conversion of microglia from a pro-inflammatory phenotype to an anti-inflammatory phenotype and prevents neuronal loss after SCI. Furthermore, we demonstrated that reducing α-Syn can improve BCSB function and astrocyte activation after SCI, reduce the occurrence and development of neuroinflammation, and confer a neuroprotective immune microenvironment. This study provides a basis for future research on immune regulation after SCI with regard to α-synucleinopathy.

## Supplementary information

**Additional file 1. **Lentiviral-SNCA-shRNA transfection efficiency and validation (*in vitro* experiments)

**Additional file 2:.** Expression of glial cells at the injury site on the first day after SCI. (A-B) Representative images showing Iba1+ staining after SCI by IHC. Scale bars = 250 μm, 100 μm. (C-D) Representative images showing GFAP+ staining after SCI by IHC. Scale bars = 250 μm, 100 μm. (E) Quantitative analysis of the mean IOD of Iba1 in panel A, n = 5. (F) Quantitative analysis of the mean IOD of GFAP in panel C, n = 5. All data are presented as the mean ± SD. One-way ANOVA and Tukey’s multiple comparisons test were used to analyse differences among groups

## Data Availability

The datasets used and/or analysed during the current study are available from the corresponding author on reasonable request.

## Abbreviations

Arg1: Arginase-1
BBB: Basso-Beattie-Bresnahan
BCSB: Blood-cerebrospinal barrier
CNS: Central nervous system
CNTF: Ciliary neurotrophic factor
CSF: Cerebrospinal fluid
ELISA: Enzyme-linked immunosorbent assay
GCRP: G-coupled receptor protein kinase
GFAP: Glial fibrillary acid protein
GM: Grey matter
HE: Haematoxylin-eosin
Iba1: Ionized calcium-binding adaptor molecule 1
IFN: Interferon
IL: Interleukin
iNOS: Inducible nitric oxide synthase
IOD: Integrated optical density
KD: Knockdown
LFB: Luxol fast blue
LV: Lentivirus
MAPK: Mitogen-activated protein kinase
MMP: Matrix metalloproteinase
NF-κB: Nuclear factor kappa B
RT-qPCR: Quantitative real-time polymerase chain reaction
SCI: Spinal cord injury
shRNA: Short hairpin RNA
TGF: Transforming growth factor
TNF: Tumour necrosis factor
WM: White matter
α-Syn: α-Synuclein
