# Supplementary material for: Lentivirus-mediated downregulation of α-synuclein reduces neuroinflammation and promotes functional recovery in rats with spinal cord injury
Source: J Neuroinflammation. 2019 Dec 30;16:283. doi: 10.1186/s12974-019-1658-2 (PMC6936070; doi:10.1186/s12974-019-1658-2)
Supplement: Supplementary file 1 — Additional file 1. Lentiviral-SNCA-shRNA transfection efficiency and validation (in vitro experiments) [file 12974_2019_1658_MOESM1_ESM.pdf]

Additional file 1

Gene	Sample	Sequence
Rattus Norvegicus SNCA	shRNA1	GCAGTGAGGCTTATGAAATTTCAAGAGAATTTCATAAGCCTCACTGCTTTTTT
	shRNA 2	CGTTCATGGAGTGACAACAGTTTCAAGAGAACTGTTGTCACTCCATGAACGTTTTTT
	shRNA 3	GCCAAAGAGCAAGTGACAAATGTTCAAGAGACATTTGTCACTTGCTCTTTGGTTTTTT
	shRNA 4	GACTATGAGCCTGAAGCCTAATTCAAGAGATTAGGCTTCAGGCTCATAGTCTTTTTT

Supplementary Table 1

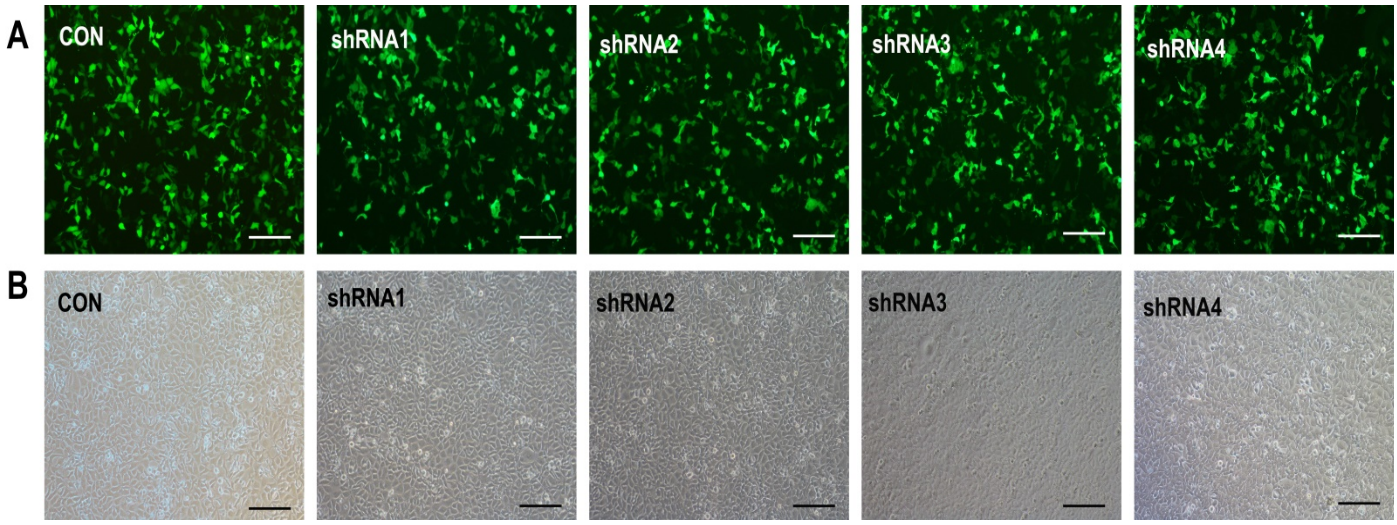

Supplementary Figure 1

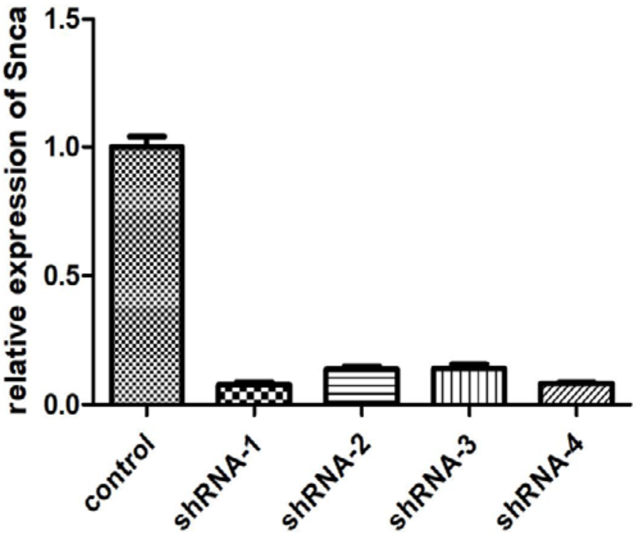

Supplementary Figure 2

Additional file 1: We designed a total of 4 sets of SNCA knockout sequence plasmids with GFP fluorescent tags, integrated them into lentiviruses and transfected them into HEK293 cells to observe the degree of transfection. qRT-PCR experiments were then performed.

Supplementary Table S1. Forward and reverse primer sequences for 4 sets of SNCA knockout sequences used for qRT-PCR.

Supplementary Figure S1. The expression pattern of SNCA in different samples of HEK293 cells.

(A) The expression levels of GFP-tagged sequences in different samples of HEK293 cells. Scale bar =100 μm.

(B) The expression levels of samples without GFP in HEK293 cells under bright-field illumination. Scale bar =100 μm.

Supplementary Figure S2. The relative expression levels of SNCA in different samples.
