# Supplementary figures and images for: Lentivirus-mediated downregulation of α-synuclein reduces neuroinflammation and promotes functional recovery in rats with spinal cord injury
Source: J Neuroinflammation. 2019 Dec 30;16:283. doi: 10.1186/s12974-019-1658-2 (PMC6936070; doi:10.1186/s12974-019-1658-2)

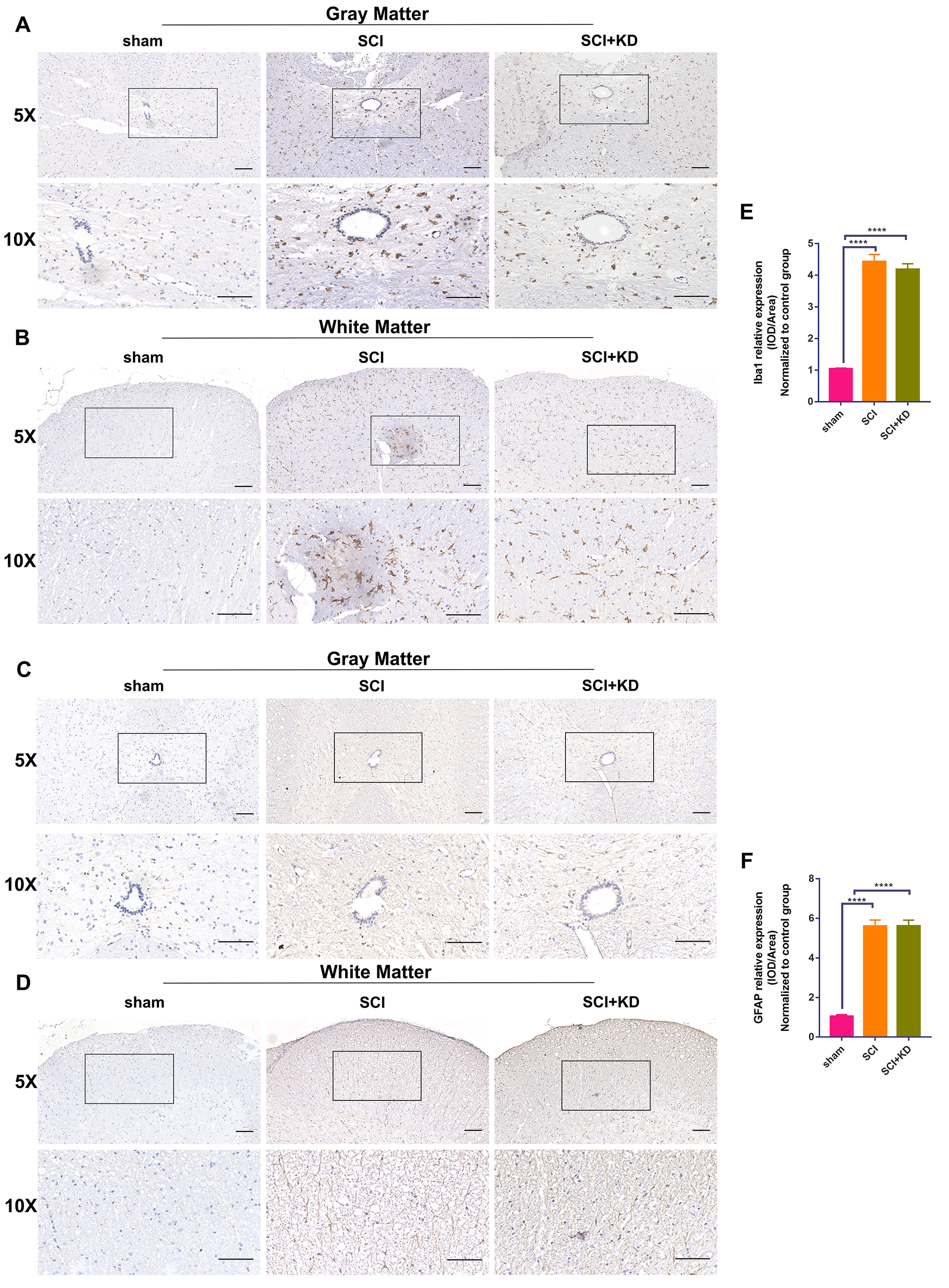

Supplement: Supplementary file 2 — Additional file 2:. Expression of glial cells at the injury site on the first day after SCI. (A-B) Representative images showing Iba1+ staining after SCI by IHC. Scale bars = 250 μm, 100 μm. (C-D) Representative images showing GFAP+ staining after SCI by IHC. Scale bars = 250 μm, 100 μm. (E) Quantitative analysis of the mean IOD of Iba1 in panel A, n = 5. (F) Quantitative analysis of the mean IOD of GFAP in panel C, n = 5. All data are presented as the mean ± SD. One-way ANOVA and Tukey’s multiple comparisons test were used to analyse differences among groups [file 12974_2019_1658_MOESM2_ESM.tif]
